# From Evaluation to Prediction: Behavioral Effects and Biological Markers of Cognitive Control Intervention

**DOI:** 10.1155/2020/1869459

**Published:** 2020-02-24

**Authors:** Bin Xuan

**Affiliations:** School of Educational Science, Anhui Normal University, Wuhu 241000, China

## Abstract

Although the intervention effectiveness of cognitive control is disputed, some methods, such as single-task training, integrated training, meditation, aerobic exercise, and transcranial stimulation, have been reported to improve cognitive control. This review of recent advances from evaluation to prediction of cognitive control interventions suggests that brain modularity may be an important candidate marker for informing clinical decisions regarding suitable interventions. The intervention effect of cognitive control has been evaluated by behavioral performance, transfer effect, brain structure and function, and brain networks. Brain modularity can predict the benefits of cognitive control interventions based on individual differences and is independent of intervention method, group, age, initial cognitive ability, and education level. The prediction of cognitive control intervention based on brain modularity should extend to task states, combine function and structure networks, and assign different weights to subnetwork modularity.

## 1. Introduction

Cognitive control, also known as executive function, is an indispensable and comprehensive ability in daily life [1, 2]. Many complex cognitive processes, including attention, memory, language, reasoning, and decision-making, require cognitive control to eliminate interferences, suppress inappropriate responses, adapt flexibly to the environment, adjust plans, and update and transform information in a timely manner [3, 4]. Cognitive control therefore plays a highly significant role in daily life.

Cognitive control deficits have been identified in children with developmental abnormalities and in adults with cognitive aging, brain damage, and a variety of neurological and psychiatric disorders. Therefore, researchers have increasingly focused on developing noninvasive methods to improve cognitive control functions; these include a specific task training of various durations and frequencies, integrated training, games, aerobic exercise, mindfulness meditation, and transcranial electric or magnetic stimulation methods.

## 2. Evaluation of the Effects on Cognitive Control Interventions

### 2.1. Indicators of Intervention Gains in Cognitive Control

Intervention often results in changes in behavior as well as the brain itself due to its flexibility or plasticity [5]. Plasticity denotes the potential modifiability of a person's cognitive abilities and brain activities [6]. Lovden et al. (2000) proposed that adult cognitive plasticity is driven by a prolonged mismatch between functional organismic supplies and environmental demands and denotes the brain's capacity for anatomically implementing reactive changes in behavioral flexibility. Plasticity typically requires substantial amounts of time and efforts, as it needs to overcome the inherent inertia of biological cognitive systems, so plastic changes (i.e., functional changes with structure changes) are often more sluggish than changes based on flexibility (i.e., just performance and functional changes) [7].

The extent to which cognitive control can be affected by training or other means is an important question, as is the question of which criteria should be adopted to evaluate the effects and efficacy of a chosen intervention. Since cognitive control intervention may affect functional changes both with and without structural alterations [8–12], in this review, we do not strictly distinguish the intervention gains derived from plasticity or flexibility. We will discuss intervention improvements at three levels. Take a specific training task as an example. The first level is the behavioral performance on the trained task; the second level is the transfer effects that generalize the trained task to near or far cognitive abilities; and the third level is the functional, network, and structural changes in the brain.

More importantly, if individual differences can be fully considered and intervention effects can be predicted based on individual behaviors or biological characteristics, this will help clinicians to select the most suitable intervention methods, thus allowing for more efficient improvement in the cognitive control of individuals [13].

### 2.2. Behavioral Improvement and Transfer Effects Induced by Interventions

Cognitive control is a comprehensive ability that contains several complex components. According to the classical theory, cognitive control includes at least three main components: inhibition control, working memory, and task switching [14, 15]. Therefore, the most direct intervention involves conducting a single-task training for a certain component, investigating the validity of the intervention by manipulating the training task, duration, and intensity, and comparing the response times and accuracy before and after training. The task performance after training usually improves to some extent, but the improvement varies with the task type, duration, and strength. For inhibition control, the training tasks usually include the Stroop Task, Flanker Task, Stop-Signal task, and Go/No-go Task [16–18]; the N-Back or working memory updating tasks are often used as training tasks for working memory [19, 20]. The component of task switching is trained by the alternating-runs paradigm and the task-cueing paradigm [21, 22]. The training stage may last 15 minutes to more than an hour for five days to five weeks. However, the length of a training does not seem to be a crucial factor in determining its efficacy, meta-analyses of executive control and working memory training in older adults suggested that gains did not vary with total training time [6, 23].

If the performance of a training task is the only aspect that improves, or if the behavioral improvement is not transferred to similar tasks, tasks with different components, or real life situations, then the significance of training is too narrow [24]. Most training claims follow from the assumption that practice yields improvements that go beyond the practiced tasks. Therefore, the evaluation of cognitive control intervention must consider the transfer effect based on differences in distance. Transfer distance refers to the taxonomy describing similarities in the nature of, and processes that underlie, outcome measures compared to the trained tasks [5]. Generally, training studies categorize outcome measures in terms of trained outcomes (or criterion tasks), near-transfer outcomes (untrained tasks similar to, but measuring the same construct as, the trained task), or far-transfer outcomes (tasks measuring a different cognitive construct) [25]. In terms of cognitive control training, near-transfer, such as the transfer from response inhibition to cognitive inhibition, and far-transfer, such as the transfer from inhibition control to fluid intelligence, are often used to evaluate the training effect [26, 27].

Karbach and Verhaeghen examined the effects of process-based cognitive control training in older adults; their interventions resulted in promising transfer of the training effects, as significant medium to small-sized effects were observed in the performance of the trained task, near-transfer tasks, and far-transfer tasks [6]. However, studies have also shown that the transfer effect decreases with increasing distance between two tasks [24, 28–31]. Meta-analyses of transfer effects on cognitive control among children, young and older adults suggested a significant near-transfer effect and no convincing evidence of far-transfer [24, 29, 32]. The evidence from young adults indicates that working memory training did not elicit any improvements across all ranges of transfer [33], but produced short-term, specific training effects that do not generalize to measures of “real-world,” such as intelligence, life ability, or academic performance [27, 32]. These arguments suggested that there may be limitations in training only for certain components or tasks in cognitive control interventions.

Since transfer effect is an important index of the efficacy of an intervention, intervention research has developed several ideas for how to obtain a better transfer effect in cognitive control. According to the inconsistent results and possible limitations of single-task training, the degree to which a training task is extensive corresponds to the likelihood that it will be transferred to other tasks or general cognitive control components. Therefore, some studies have used computerized cognitive training (CCT) or customized video games, such as NeuroRacer [34], Advanced Cognitive Training for Independent and Vital Elderly (ACTIVE) [35], or commercial cognitive training products such as Cogmed [28]. Several studies with meta-analysis indicate that CCT or video game training produce positive but modest effects on improving several aspects of cognitive performance in healthy young and older adults [36–38].

Compared with the single-component training task, tasks that train multiple cognitive control components are more complex and have resulted in improved performance that was also transferred to broad cognitive processes of daily living abilities such as driving behavior [34, 39]. However, Simons et al. reviewed intervention effects from products of Lumos Labs, Posit Science, Nintendo, and Cogmed and found extensive evidence that interventions improved performance on the trained tasks, less evidence that such intervention improved performance on closely related tasks, and little evidence that training enhanced performance on distantly related task or everyday cognitive ability [40]. Similar conclusion was also reached in study by Owen et al. (2010) that no evidence was found for transfer effects to untrained tasks, even when those tasks were cognitively closely related [24].

Diverse activities have been shown to improve executive function of children 4–12 years old, including computerized training, noncomputerized games, aerobics, martial arts, yoga, mindfulness, and school curricula [41]. The best evidence exists for computer-based training, traditional martial arts, and two school curricula [42]. Diamond and Lee [41] suggested that to improve executive functions, focusing narrowly on them may not be as effective as also addressing emotional, social, and physical development.

Recent studies have explored the effects of interventions by physical exercise. Xue et al. reviewed the effects of chronic exercise in healthy children aged 6 to 12 years and adolescents aged 13–17 years. The results showed that chronic exercise interventions improved executive functions [43]. A small facilitating effect of high-intensity exercise on cognitive control was also found in children, and the effect was not different from low-to-moderate intensity exercise [44].

The intervention effect of physical exercise has also been investigated on young and older adults. In healthy young adults, acute aerobic exercise, such as 30 minutes of self-paced motor-driven treadmill exercise at 70% intensity of maximum heart rate, may serve to facilitate the flexibility of task-set reconfiguration and maintain the task set in working memory [45]. A study of 20-day dual n-back training following aerobic exercise on young males revealed weak-to-moderate evidence for exercise-induced facilitation on cognitive training; the combination of cognitive training with exercise resulted in greater transfer gains on conditions involving greater attentional demanding [46].

Colcombe and Kramer found that older adults who participated in a 6-month aerobic training program showed robust but selective benefits for cognition, with the largest aerobic fitness-induced benefits occurring for executive control processes [47]. In addition, the meta-analysis indicates just walking can improve set-shifting and inhibition in sedentary older persons without cognitive impairment [48]. However, Diamond et al. (2019) suggested that aerobic exercise interventions, resistance training, and yoga have produced the weakest results for improving cognitive control.

Transcranial direct current stimulation (tDCS) is believed to modulate cognition in healthy adult population, but a quantitative review does not support the idea that tDCS generates a reliable effect on executive function in healthy adults [49]. There is little evidence that tDCS alone improves working memory and related cognition [50, 51]. In contrast, the more consistent evidence supported that anodal tDCS coadministered with cognitive tasks can significantly enhance cognitive performance [51–53]. The integration of different approaches seems to be more useful for cognitive control intervention [54, 55]. Huo et al. (2018) found that tDCS independent of cognitive training did not show a beneficial effect on executive function for healthy older adults, presumably because the effect of the stimulation lies in its amplification of training gains. These findings also indicate that combining traditional cognitive training methods with brain stimulation may be a better approach for improving executive function, may result in better transfer effects, and may even enhance fluid intelligence [26, 56, 57].

Moreover, recent studies have pointed out that the ultimate goal of cognitive control is to reduce uncertainty [4, 58]. In Shannon's information theory, information is defined as entropy, and uncertainty is quantified as the information entropy in units of bits for a given channel [4]. In an event sequence, if events are predictable, the uncertainty of the events is low. For example, Go/No-Go performance involves examining responses to a stimulus type with a low probability of occurrence in a series of high probability events. Probabilities for Go and No-Go trials can be assumed to be 0.80 and 0.20, respectively. The difference in information conveyed by the occurrence of Go and No-Go events can be quantified as the 2-bit difference between these two surprise values. This is a novel and comprehensive account of cognitive control, which treats the brain as an information-processing entity wherein cognitive control plays a pivotal role in dealing with conditions of uncertainty [59]. According to the theory of cognitive control, conflict across different paradigms used in the study of cognitive control is only one type of uncertainty increase. Thus, if training tasks are designed to reduce uncertainty, and an intervention targets the essence of cognitive control, it should be possible to improve the efficiency of the intervention and broaden its transfer effect.

### 2.3. Changes in Brain Regions Induced by Interventions

Behavioral changes after cognitive control interventions are often accompanied by changes in brain activity and even brain structures [60]. Structurally, the possible benefits of different interventions include delays in thickness atrophy of the prefrontal cortex [61, 62], an increase in white matter integrity [63, 64], and increased volume of white and gray matter [65–67].

Intervention-related changes are more strongly reflected in changes in brain activity during task and resting states. Some studies have found that a cognitive control intervention led to decreased activation of the frontal lobe in elderly participants under medium- and low-difficulty conditions and to decreased theta wave energy in the frontal lobe under interference conditions; this suggests that the training improved neural efficiency [20, 57, 68, 69]. Similar effects have also been reported after long-term aerobic exercise, whereby individuals showed a decreased activation of the frontal lobe and an increased activation of the hypothalamus and striatum in the Flanker task [57, 70]. The amplitude of low frequency fluctuations (ALFF) has also been reported to show high reliability in the evaluation of intervention effects. For older adults, six weeks of an integrated intervention enhanced the ALFF of the superior frontal gyrus and medial frontal gyrus, and individual differences based on the ALFF were related to postintervention behavioral changes [71].

Moreover, the transfer effect of training has been found to have a corresponding neural basis. Millner et al. found that for young adults, training reduced response time and decreased the N2 amplitude under inconsistent conditions [30]; moreover, the N2 origin was sourced to the dorsal anterior cingulate cortex (dACC) [72, 73]. Some studies have also found that activation of the dACC and dorsal lateral prefrontal cortex (DLPFC) decreased after training [74, 75]. These results suggest that training may promote the efficiency of the dACC or alter the connection between the dACC and DLPFC, thereby reducing the conflict effect. Dahlin, et al. found that behavioral transference only occurred in participants who exhibited increased activation of the striatum after training [76]. Furthermore, the behavioral transfer effects were accompanied by an enhanced energy of the midline brain and posterior frontal lobe [34]. A shared neural basis between the training and transfer tasks, such as the lateral prefrontal cortex, is likely the neural basis of the transfer effect [77].

### 2.4. Changes in the Brain Network Connectivity Induced by Interventions

Changes brought about by cognitive control interventions include not only the activation of a single brain region or the change of a specific electroencephalogram (EEG) characteristic but also changes in the strength and patterns of connectivity between brain regions in a large-scale network, which may involve larger ranges and subtler changes. The benefits of four or more weeks of cognitive training have been reported to include increased functional connectivity within the frontoparietal network, increased global and local blood flow of the default and execution networks, and enhanced network connection [64, 78, 79].

Meditation is also thought to improve cognitive control by changing the state of the brain's network connections. Through methods such as mindfulness meditation, individuals' overall attention state can be autonomously adjusted to promote their metacognition and cognitive flexibility, which improves cognitive control [8, 80, 81]. This ability is based on the voluntary control of attentional focus, and it involves maintaining attention on the immediate experience, away from distractions such as self-referential thinking and mind wandering [82]. At the neural level, the prefrontal cortex (PFC) is one of the brain regions that plays a central role in the top-down control of information processing [83]. Previous studies showed that mindfulness practices are associated with increased PFC activation and decreased amygdala activations [84–86]. In addition, meditation has been reported to reduce the connections between the default and salience networks and between the default and frontoparietal networks; to increase the connections between the posterior cingulate cortex, the medial prefrontal lobe, and the left hippocampus; and to maintain the continuous effect on controlling the default mode network (DMN). The balance of the activation and deactivation of the DMN appears to be important in maintaining healthy brain function, including executive function, memory, and attention, and meditation training has been shown to alter patterns of brain activity of DMN and TPN, which can be used as a strategy for neuroprotection [82].

## 3. Prediction of the Effects on Cognitive Control Interventions

### 3.1. Intervention Effects of Cognitive Control across Different Populations

Participants in previous cognitive control interventions have included children, adolescents, young and older adults [17, 23, 30, 87], and some special groups, such as patients with depression, schizophrenia, impulsive disorder, or ADHD [88–90]. Of particular concern is intervention for older adults. Existing studies have shown that the brains of older adults maintain a certain degree of plasticity, which through cognitive control training can delay aging related cognitive control. Through training on working memory, attention, and goal management, most studies have shown that cognitive control interventions for older adults are effective. Computerized cognitive training (CCT) can improve the participation of the subjects, adjust the training difficulty according to the performance of the subjects, which can lead to a better effect of the intervention [91]. In addition, multidomain training is superior to single-field cognitive training [92]. Multidomain cognitive training combined with aerobic exercise, mindfulness meditation, lifestyle changes, or physical stimulation may be a more effective channel to improve the effectiveness of the intervention and increase the transfer effect.

Depression is characterized by disordered affect and difficulties in emotion regulation, and patients with depression can also show impaired cognitive control [93]. Therefore, cognitive control training is widely used for as an intervention for depression [94]. Studies have shown that single-session cognitive control training or multisession adaptive Paced Auditory Serial Addition Task (PASAT) training can reduce the cognitive sensitivity of patients with depression [95–97]. In addition, participants undergoing concurrent cognitive control training and tDCS were characterized by heightened cognitive control over negative stimuli. Interestingly, improved cognitive control over negative stimuli was associated with lower ratings of depression severity [98]. This indicates that the use of neurostimulation techniques or computerized training tasks has a beneficial effect on depressive symptomatology directly following training, and that in the long-term, patients might even benefit from a combined approach.

### 3.2. Intervention Effects of Cognitive Control Based on Individual Cognitive Profiles

The purpose of predicting intervention effects based on individual characteristics is to ascertain “who” benefits and in “which task” that person benefits from the intervention [23, 87]. The answers for “who” and “which task” may be helpful for designing the most effective training to suit an individual's cognitive profile. Several factors, such as age, general cognitive ability, baseline performance of the trained task, and formal education, are often believed to have roles as predictors and modulators of the intervention benefits [99]. Previous studies have found that the effects of individual factors are often interpreted in two seemingly opposite directions: the compensation effect (high-performing individuals will benefit less from the training) and magnification effects (high-performing individuals will benefit more from the training) [87, 100–102].

In fact, data on the role of an individual's cognitive profile in training-related performance gains are rather mixed. Borella et al. found that the role of individual characteristics depended on the type of measure examined, and effects of these variables were very modest for some tasks in older adults. In general, the more the tasks demanded active information processing, the more the factors examined seemed to support a magnification effect. That is, participants who had a good profile (i.e., younger participants or those with higher baseline performance) were more likely to improve after the training. In contrast, for more passive tasks, the results supported a compensation effect: participants with lower cognitive profile benefited more from the training [23]. Given the complexity of prediction, it is crucial to find reliable indicators for predicting intervention effects across populations and individual cognitive backgrounds. The results and discussion may be presented separately, or in one combined section, and may optionally be divided into headed subsections.

### 3.3. The Relationship between Modularity and Prediction of Cognitive Control Plasticity

Cognitive control is a relatively complex function that cannot be confined to a single brain region, and instead relies on the wider communication between distributed brain networks. Thus, it seems to be more effective to predict cognitive control functions through some features of the brain network [103, 104]. Topological properties of brain network structures, such as modularity, layering, centrality, and distribution of network central nodes, play an increasingly important role in our understanding of the complex human brain [105]. Recent studies have shown that the modularity of brain networks may be a biological marker of cognitive control plasticity, which can not only be used to evaluate the effects of cognitive control interventions but can also provide better predictions to help individuals make more informed clinical decisions regarding intervention choices [106, 107].

**Figure 1:**
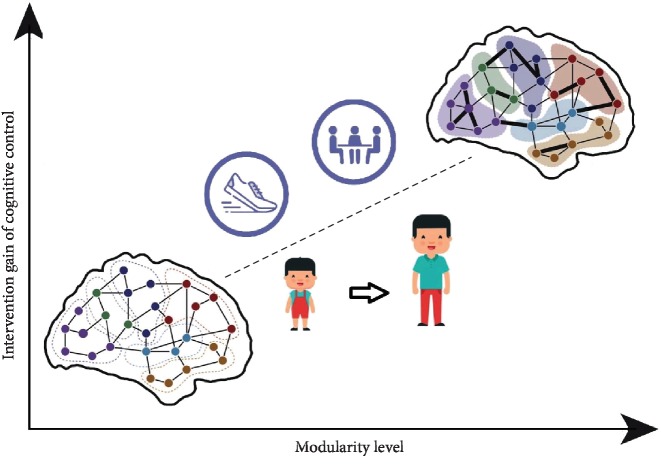
The relationship between modularity and cognitive control influenced by intervention and development. Adapted from Baum et al. [108] and Gallen and D'Esposito [105].

The relationship between brain modularity and cognitive control changes with individuals' development and aging. As individuals develop and mature, brain modularity exhibits trends in which the intermodule connections weaken, the intramodule connections become stronger, and modules develop more distinct boundaries and become more independent [108]. This reduces the interference between different networks and promotes the specialization of brain functions. The separation of modules is consistent with the differences seen in cognitive control function in individual development. During adolescence, cognitive control tends to increase with age, and brain modularity gradually increases. As an individual ages, cognitive control functions begin to decline, and the modular characteristics of the brain gradually weaken [109]. Similarly, after cognitive control interventions, modularity is further enhanced; thus, modularity seems to be an effective biological indicator of cognitive control improvement. It is worth noting that there is a very consistent relationship between modularity and cognitive control improvement induced by physical development and intervention (Figure 1).

Interindividual differences in brain modularity can be a stable indicator for predicting the effects of cognitive control interventions. Previous studies have shown that differences in the behavior performance, volume, or activation of single brain regions seem to predict differences in improvement of cognitive control [87, 110, 111]; however, when the prediction was applied to different groups or different intervention methods, the consistency of the prediction still needed to be improved, and neurobiological markers at the level of brain networks may be more reliable.

### 3.4. Prediction Stability across Intervention Methods and Populations

Modularity has shown considerable consistency and stability across populations and intervention methods as a predictor of cognitive control intervention effects. After five weeks of a mindfulness intervention focusing on attention regulation and practice in daily life, patients with chronic brain injury showed behavioral improvements in subsequent cognitive control tasks. Specifically, patients with a high level of brain modularity experienced a higher cognitive benefit from training [112]. Another study with healthy older adults also found that individuals with high modularity showed a greater performance improvement after 12 weeks of cognitive training [113]. Intervention studies on cognitive control have primarily focused on CCT and group interventions [9]; however, exercise training has also been reported to have positive effects on brain function and behavioral performance. Consistent with findings from cognitive training and group interventions, higher levels of brain modularity were associated with greater improvements in exercise-related cognitive control [10, 114].

On reviewing studies that have investigated the relationship between modularity and cognitive control benefits, modularity was found to be a very stable predictor across populations (from patients with brain damage to healthy individuals) and intervention methods (e.g., CCT, group mindfulness interventions, aerobic exercise), and it was not influenced by educational background, age, initial cognitive ability, or brain volume. The stability of modularity as a predictor was not affected by individuals' states in the resting state scanning or the data analysis methods [113]; this suggests that, as an independent factor, modularity can adequately predict the benefits that individuals will derive from cognitive control interventions [105, 112, 113].

## 4. Limitations of the Cognitive Control Interventions

The premise for exploring effective methods of improving cognitive control is the belief that cognitive control has a certain behavioral and neural plasticity that can be trained and modified. However, previous studies have shown that not all training methods effectively enhance cognitive control [24] or improve performance on closely related tasks or on distantly related tasks [19, 40], and genetic research has raised further doubts regarding cognitive control plasticity.

Heritability is generally thought to be the proportion of variance that can be attributed to genetic rather than strictly environmental factors. Generally, heritability is estimated by comparing monozygotic and dizygotic twins [115]. Fan et al. investigated 26 pairs of homozygotic and heterozygotic twins aged 14 to 42 years old and found that the heritability of executive control components was as high as 72% in an attentional network task [115]. In another study with 78 pairs of homozygotic twins and 80 pairs of heterozygotic twins, a series of cognitive control tasks were performed; after excluding the influence of age and education, there was a significant genetic effect for each task, including a heritability of 79% of the shared components of cognitive control [116]. These findings suggest that there is very little room for intervention effects on cognitive control.

From the perspective of behavioral genetics, Friedman et al. systematically explored the relevance of the three main components of cognitive control: inhibiting dominant response, updating working memory representations, and shifting between task sets [117]. The authors found that these components were affected by common factors of up to 99% heritability, which is far beyond the influence of intelligence and perceptual speed. Geschwind et al. showed that the heritability of the frontal lobe volume ranged from 0.5% to 0.7% [118]. Compared with other brain regions, the surface area of the frontal lobe has the highest heritability [119]. To some extent, these results limit the further exploration of cognitive control plasticity.

Despite considerable disputes over whether cognitive control is plastic or not, and how much room there is for any effect of cognitive control interventions, there still have been significant attempts to improve cognitive control through different methods and over various time periods [8, 34, 120]. The results of these studies showed that cognitive control was not always unchangeable, and numerous interventions effectively improved this ability [6, 34, 60]. This challenges the idea that cognitive control cannot be modified and offers new ideas for the evaluation and prediction of intervention effects. Therefore, based on the confirmation of intervention validity, researchers should identify effective indicators to evaluate and predict plasticity of cognitive control, as well as consider the influence of various factors on the intervention effects. These factors include adaptivity, strength of the intervention, expectation and motivation of the participants, and other individual differences which could significantly influence the assessment of the validity of an intervention [9]. For example, adaptivity is defined as the modification of stimuli or responding characteristics of the challenge as determined by an individual's performance, and is often assumed to be central to an optimal training experience [9]. Adaptivity intervention is frequently employed in video games involving working memory [87] and inhibition control [121], to enhance the playing experience with slow increase in levels' difficulty to encourage subsequent play.

## 5. Conclusions

The value of evaluating and predicting cognitive control plasticity lies in the consideration of individual differences and the development of more effective and targeted intervention methods that can be applied in clinical practice and daily life. While behavioral improvements are still the final criteria for evaluating the effects of cognitive control interventions, changes in brain structure and function may support these behavioral effects. Previous studies have evaluated intervention efficacy at three levels. First, the improvement of behavioral performance is investigated. Second, the temporal extension of the intervention effects is considered; in addition to the immediate effects obtained from short-term measurements, the delay effect is also tracked, and the maintenance of intervention effects is investigated [87, 122]. The third level considers the transfer effect and generalization of intervention effects to other tasks and daily life, which highlights the ecological validity of cognitive control interventions [123]. In future studies, evaluation data could be used to train the essential or general components of cognitive control to achieve a better transfer effect and longer-lasting benefits. It should be noted that when evaluating the effects of cognitive control interventions, a standardized active control group should also be manipulated to increase the reliability.

Concerning the prediction of the effects of cognitive control interventions, existing research has shown that modularity can be regarded as an independent predictor that is unaffected by age, educational level, and basic cognitive ability. However, previous studies have often focused on modularity during a resting state. Future research should further consider individuals' modularity in a state of performing cognitive control tasks, since the task state is helpful in reducing individual involuntary movement and to improve predictability [124]. Furthermore, previous studies have often investigated modularity of functional and structural networks independently. In future work, corresponding biological markers should be extracted from the relationships between the two types of networks.

Finally, previous studies often used modularity of the whole brain as the indicator; however, existing studies on brain injury have shown that the modularity of each network differs in its predictive ability of the effects of cognitive control interventions. In contrast to sensorimotor networks, the modularity of association networks, such as the frontoparietal network, may better predict the benefits of cognitive control training or neuroplasticity. Therefore, to assign different weights to modularity of subnetworks in the prediction could be helpful for predicting what individuals will benefit from cognitive control interventions.
